# Glyceryl Trinitrate Enhances Caffeine Cytotoxicity Under Metabolic Stress in Cancer Cells

**DOI:** 10.3390/molecules31111946

**Published:** 2026-06-04

**Authors:** Vesna Zeljković, Mirjana Bogavac, Tanja V. Soldatović, Marko Mladenović, Zoran Marković, Elvis Mahmutović, Maja Karaman

**Affiliations:** 1 Department of Biomedical Sciences, State University of Novi Pazar, Vuka Karadžića 9, 36300 Novi Pazar, Serbia; ehmahmutovic@np.ac.rs; 2 Department of Obstetrics and Gynecology, Faculty of Medicine, University of Novi Sad, Hajduk Veljkova 3, 21000 Novi Sad, Serbia; mirjana.bogavac@mf.uns.ac.rs; 3 Department of Sciences and Mathematics, State University of Novi Pazar, Vuka Karadžića 9, 36300 Novi Pazar, Serbia; tsoldatovic@np.ac.rs (T.V.S.); mmladenovic@np.ac.rs (M.M.); zmarkovic@uni.kg.ac.rs (Z.M.); 4 Department of Chemistry, Faculty of Sciences and Mathematics, University of Niš, Višegradska 33, 18000 Niš, Serbia; 5 Department of Biology and Ecology, Faculty of Sciences, University of Novi Sad, Trg Dositeja Obradovića 3, 21000 Novi Sad, Serbia

**Keywords:** glyceryl trinitrate, nitroglycerin, 2-deoxy-D-glucose, caffeine, cancer metabolism, cytotoxicity, synergistic effect, Chou–Talalay method, molecular docking, ALDH2

## Abstract

Cancer cell metabolism represents a critical therapeutic target, particularly under conditions of metabolic stress induced by glycolysis inhibition. Nitroglycerin (glyceryl trinitrate, GTN), a nitric oxide donor, and 2-deoxy-D-glucose (2-DG), a glycolysis inhibitor, have individually demonstrated anticancer potential through modulation of cellular metabolism and redox balance. In this study, we investigated the cytotoxic and combined effects of GTN and caffeine under 2-DG-induced metabolic stress in human cancer cell lines (*HeLa*, *A549*, *HT29*, and *MRC-5*). Cell viability was assessed using the sulforhodamine B assay after 24 and 48 h treatments, while drug interactions were evaluated using the Chou–Talalay method and combination index (CI) values. 2-DG alone reduced cell viability in a concentration- and time-dependent manner, with IC_50_ values ranging from 2.01 to 7.05 mM depending on the cell line and exposure period. The combined treatment further enhanced cytotoxicity, particularly in *A549* cells, where viability decreased to approximately 63% after 48 h and the calculated IC_50_ value for GTN in the presence of caffeine reached 0.143 μM. CI analysis demonstrated synergistic interactions in *HeLa* and *A549* cells (CI < 1), whereas *HT29* cells predominantly exhibited antagonistic responses (CI > 1). However, strong synergistic effects were also observed in MRC-5 fibroblasts, indicating limited selectivity toward cancer cells. Molecular docking suggested favorable in silico binding of GTN to aldehyde dehydrogenase 2 (ALDH2) and caffeine to the adenosine A2A receptor. Nevertheless, these findings should be considered exploratory and hypothesis-generating because target expression, enzymatic activity, and pathway activation were not experimentally validated. Overall, the results suggest that GTN enhances caffeine-induced cytotoxicity under metabolically stressed conditions through combined metabolic and redox perturbation, although the magnitude of the response depends on cellular context and warrants further mechanistic investigation.

## 1. Introduction

Nitroglycerin (glyceryl trinitrate, GTN) is a well-established nitric oxide (NO) donor widely used as a vasodilator in the treatment of cardiovascular diseases, including angina pectoris and hypertension [1]. Beyond its clinical application, GTN has attracted attention in cancer research due to its concentration-dependent dual biological effects. At low NO concentrations (<100 nM), GTN may promote tumor progression through enhanced angiogenesis, increased cellular proliferation, and inhibition of apoptosis. In contrast, at higher NO concentrations (>500 nM), GTN induces cytotoxic effects, including oxidative and nitrosative stress, DNA damage, and apoptosis [2,3]. Previous studies have demonstrated that low to intermediate NO concentrations (approximately 100–500 nM) may stimulate tumor cell proliferation and angiogenesis, whereas higher NO levels induce oxidative stress, DNA damage, and apoptosis [2,3,4]. These antitumor effects are primarily mediated by NO-dependent mechanisms involving inhibition of cellular respiration, modulation of iron metabolism, and activation of p53-dependent apoptotic pathways [5]. Since nitric oxide signaling and metabolic regulation are closely interconnected in cancer cells, compounds targeting tumor bioenergetics, such as 2-deoxy-D-glucose (2-DG), have attracted considerable attention as potential combination partners for NO-based therapies.

2-Deoxy-D-glucose (2-DG) is a synthetic glucose analog that exerts anticancer activity primarily through the disruption of cellular energy metabolism. Following uptake via glucose transporters (GLUTs), 2-DG is phosphorylated by hexokinase to generate 2-DG-6-phosphate. Due to the absence of a hydroxyl group at the C-2 position, this metabolite cannot be further converted by phosphoglucose isomerase and consequently accumulates intracellularly. The accumulation of 2-DG-6-phosphate inhibits glycolytic flux, leading to ATP depletion, impaired bioenergetic homeostasis, and suppression of cell proliferation. Cancer cells are particularly susceptible to glycolytic inhibition because of their increased glucose consumption and dependence on aerobic glycolysis, commonly referred to as the Warburg effect [6,7].

In addition to its effects on glycolysis, 2-DG interferes with N-linked glycosylation due to its structural similarity to mannose. Disruption of protein glycosylation results in the accumulation of misfolded proteins within the endoplasmic reticulum, activation of the unfolded protein response (UPR), and induction of endoplasmic reticulum stress. Persistent ER stress may subsequently trigger apoptotic signaling pathways and contribute to tumor cell death [8,9].

Furthermore, 2-DG promotes oxidative stress by impairing mitochondrial function and reducing ATP availability, leading to increased intracellular production of reactive oxygen species (ROS). Since malignant cells frequently exhibit elevated basal ROS levels, additional oxidative stress induced by 2-DG may exceed their antioxidant capacity, thereby enhancing cellular damage and apoptosis [6,10].

A further consequence of metabolic stress induced by 2-DG is the activation of autophagy. Although autophagy initially functions as an adaptive mechanism that supports cell survival under nutrient deprivation and energy stress, prolonged activation may contribute to cell death. Several studies have demonstrated that inhibition of autophagy significantly potentiates the cytotoxic activity of 2-DG, suggesting that autophagy predominantly represents a protective response in cancer cells exposed to glycolytic inhibition [11].

Importantly, the multifaceted mechanisms of 2-DG, including inhibition of glycolysis, induction of ER stress, generation of oxidative stress, and modulation of autophagy, make this compound an attractive candidate for combination-based anticancer therapies. By targeting metabolic vulnerabilities characteristic of malignant cells, 2-DG has been shown to enhance the efficacy of chemotherapy, radiotherapy, and various targeted therapeutic approaches while exhibiting a degree of selectivity toward cancer cells compared with normal tissues [10,12].

Considering that nitric oxide signaling has been implicated in the regulation of mitochondrial respiration, oxidative stress, and autophagy, the combination of nitroglycerin and 2-deoxy-D-glucose may represent a potential strategy for targeting metabolic and redox vulnerabilities in cancer cells. This concept is supported by the enhanced cytotoxic effects observed in *HeLa* and *A549* cells following combined treatment, suggesting that metabolic stress induced by 2-DG may increase cellular susceptibility to nitroglycerin-mediated cytotoxicity.

Caffeine, a methylxanthine, modulates cellular signaling through adenosine receptor antagonism and interference with cell cycle regulation and DNA repair pathways [13,14,15,16]. It has been shown to enhance cytotoxic responses under metabolic stress and to potentiate the effects of glycolysis inhibition, thereby promoting apoptosis and reducing tumor cell viability [15,16].

Although GTN, 2-DG, and caffeine have each demonstrated individual anticancer properties, their combined effects under conditions of metabolic stress remain insufficiently explored [3,6,10,13,14,15,16,17,18]. Since GTN modulates cellular redox balance and nitric oxide signaling, 2-DG disrupts glycolytic energy metabolism, and caffeine interferes with cell cycle regulation and DNA repair pathways, their simultaneous application may produce enhanced cytotoxic and potentially synergistic antitumor effects. Therefore, the aim of this study was to investigate the cytotoxic potential of glyceryl trinitrate in combination with caffeine under metabolic stress induced by 2-deoxy-D-glucose in human cancer cell lines, with particular emphasis on interaction effects and the potential enhancement of anticancer activity.

## 2. Results

The cytotoxic effects of nitroglycerin, 2-deoxy-D-glucose (2-DG), and caffeine were evaluated in HeLa, A549, and HT29 cancer cell lines, as well as in normal lung fibroblasts (MRC-5), using the SRB assay. Cells were treated with individual agents and their combinations for 24 h and 48 h.

### 2.1. Effects of Nitroglycerin

Nitroglycerin (GTN) is an organic nitrate that acts as a prodrug and requires enzymatic bioactivation to exert its biological effects. The primary pathway of GTN bioactivation involves mitochondrial aldehyde dehydrogenase (ALDH2), which catalyzes the conversion of GTN into nitric oxide (NO) or related nitrogen reactive species. These molecules regulate multiple cellular processes, including vasodilation, mitochondrial respiration, oxidative stress, apoptosis, and autophagy. Although both normal and malignant cells possess the enzymatic machinery necessary for GTN bioactivation, cancer cells frequently exhibit altered redox balance, mitochondrial dysfunction, and dysregulated NO signaling, which may influence their sensitivity to NO-mediated cytotoxic effects. Consequently, GTN has attracted interest as a potential anticancer agent, particularly in combination with therapies targeting tumor metabolism and oxidative stress.

GTN alone exerted minimal cytotoxic effects in HeLa and A549 cells across the tested concentration range, with cell viability remaining above 85% after both 24 h and 48 h treatments. These findings suggest that GTN, as a single agent, does not significantly impair cell survival under the tested conditions, which may be related to its prodrug nature and the requirement for enzymatic bioactivation. In contrast, HT29 cells showed a moderate response, particularly after prolonged exposure, indicating cell-type-dependent sensitivity. Normal MRC-5 fibroblasts exhibited negligible sensitivity, confirming the low intrinsic cytotoxicity of GTN under these conditions.

### 2.2. Combined Treatment with 2-Deoxy-D-Glucose

**Figure 1:**
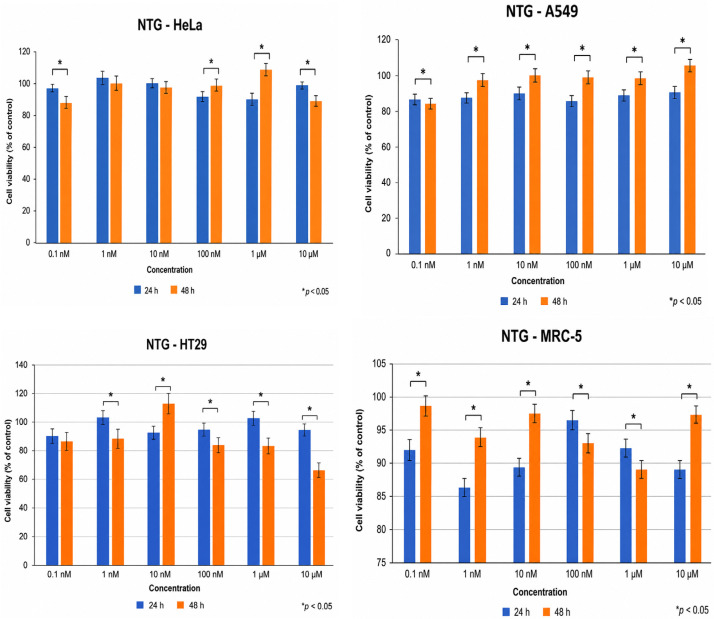
Cytotoxic activity of 2 DG in *HeLa*, *A549*, *HT29*, and *MRC-5* cells following 24 h and 48 h treatment; data are presented as mean ± SD (*n* = 3), * *p* < 0.05.

**Figure 2:**
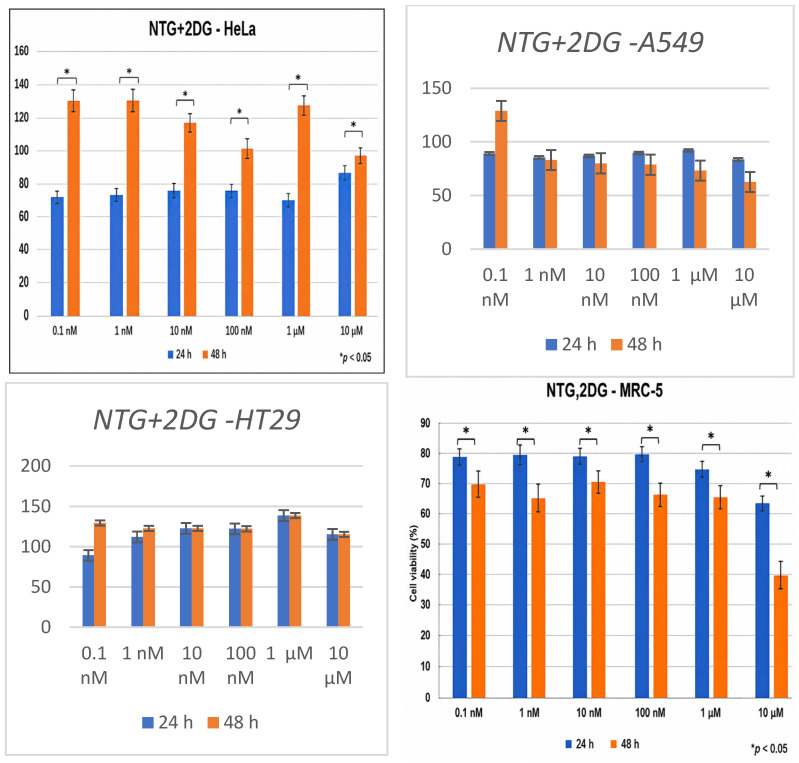
Cytotoxic activity of NTG and 2DG in *HeLa, A549*, *HT29*, and *MRC-5* cells following 24 h and 48 h treatment, data are presented as mean ± SD (*n* = 3), * *p* < 0.05.

The addition of 2-DG significantly enhanced cytotoxicity in all cancer cell lines, with the most pronounced effects observed after 48 h. As shown in Table 1, 2-DG alone exhibited concentration- and time-dependent cytotoxic effects, with IC_50_ values ranging from 2.01 to 7.05 mM depending on the cell line and incubation period. In HeLa cells, combination treatment reduced viability to approximately 59–61%, compared to >85% observed with single agents. A549 cells demonstrated a similar trend, with viability decreasing to approximately 60% at higher GTN concentrations. HT29 cells displayed a weaker response, indicating partial resistance to metabolic stress-induced cytotoxicity. Notably, MRC-5 fibroblasts also showed a substantial reduction in viability (~35%), highlighting the limited selectivity of the combination treatment. These findings suggest that glycolytic inhibition may sensitize cancer cells to GTN-mediated cytotoxicity under metabolic stress conditions, although the pronounced effects observed in normal fibroblasts indicate an important limitation for therapeutic application (Figure 1). Treatment with 2-deoxy-D-glucose induced pronounced morphological alterations characteristic of metabolic stress and cell death, including cellular shrinkage, loss of normal morphology, and reduced cell density, supporting its antiproliferative and cytotoxic activity (Figure S1). The effects of the combined treatment of NTG and 1 mM 2DG on cell viability are shown in Figure 2.

### 2.3. Combined Treatment with Caffeine

The addition of caffeine further modulated cytotoxic responses. While limited effects were observed after 24 h, prolonged exposure (48 h) resulted in enhanced cytotoxicity, particularly in *A549* cells, where cell viability decreased below 50% at higher concentrations. This finding suggests that caffeine may potentiate GTN-induced cytotoxicity, possibly through modulation of cell cycle regulation and DNA damage response pathways. A marked reduction in viability was also observed in *MRC-5* fibroblasts, further confirming the limited selectivity of the combination treatment.

*HT29* cells were the least sensitive to the combined treatment (Table S2). Cell viability remained above 60% after 24 h and above 75% after 48 h even at the highest tested concentration (10 μM GTN + 1 mM caffeine), indicating that the IC_50_ value exceeded the investigated concentration range.

These results support the hypothesis that caffeine enhances the cytotoxic response of cancer cells under metabolically stressed conditions, although additional studies are needed to clarify the underlying mechanisms.

### 2.4. Docking Experiments

Proteins 1O01 and 1O02, representing ALDH2 structures, were selected for molecular docking with nitroglycerin (GTN) based on the previously reported involvement of ALDH2 in GTN bioactivation and nitric oxide (NO)-related signaling [4,11]. Therefore, ALDH2 was considered a literature-supported target for the theoretical evaluation of GTN binding. However, it should be emphasized that the present docking analysis does not demonstrate ALDH2 expression, enzymatic activity, NO release, or pathway activation in the investigated cell lines. Accordingly, the interaction between GTN and ALDH2 should be interpreted as a theoretical and hypothesis-generating observation rather than direct experimental evidence of ALDH2-mediated bioactivation in HeLa, A549, HT29, or MRC-5 cells.

The molecular docking results obtained using CB-Dock2 (Table 2) showed that the predicted binding energies of GTN and caffeine with the selected target proteins ranged from −6.2 to −6.5 kcal/mol. Negative binding energy values indicate energetically favorable in silico ligand–protein interactions, with lower values generally corresponding to more stable predicted complexes. Nevertheless, docking scores represent theoretical estimates of binding affinity and should not be interpreted as experimental evidence of target engagement, receptor activation or inhibition, or functional pathway involvement in the tested cell lines.

Docking scores represent predicted in silico binding affinities and should be interpreted only as theoretical estimates. They do not provide direct evidence of target expression, target engagement, pathway activation, or functional involvement in the tested cell lines.

### 2.5. Chou–Talalay Analyses

**Figure 3:**
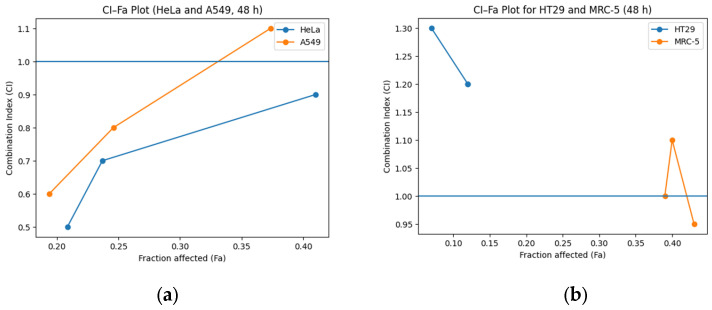
Combination index (CI) versus fraction affected (Fa) plots for the interaction between nitroglycerin (GTN) and 2-deoxy-D-glucose (2-DG) after 48 h treatment. (**a**) HeLa and A549 cells showing predominantly synergistic interactions (CI < 1); (**b**) HT29 and MRC-5 cells showing variable or antagonistic interactions; CI < 1 indicates synergism, CI = 1 indicates an additive effect, and CI > 1 indicates antagonism.

CI–Fa analysis revealed distinct interaction patterns across the examined cell lines. *HeLa* cells consistently showed synergistic effects (CI < 1) across the tested range of fraction affected (Fa) values, indicating a strong cooperative interaction between GTN and 2-DG. In *A549* cells, the interaction shifted from synergistic to nearly additive at higher effect levels, suggesting partial loss of synergy under stronger cytotoxic conditions. *HT29* cells predominantly exhibited antagonistic responses (Figure 3a), indicating reduced sensitivity to the combined treatment and possible metabolic adaptability. Variable CI values were observed in *MRC-5* cells (Figure 3b), accompanied by substantial cytotoxicity, further emphasizing the limited selectivity of the treatment toward cancer cells. The corresponding numerical data used for CI–Fa interpretation, IC_50_ comparison, and viability-based response classification are provided in Supplementary Tables S1–S4.

**Figure 4:**
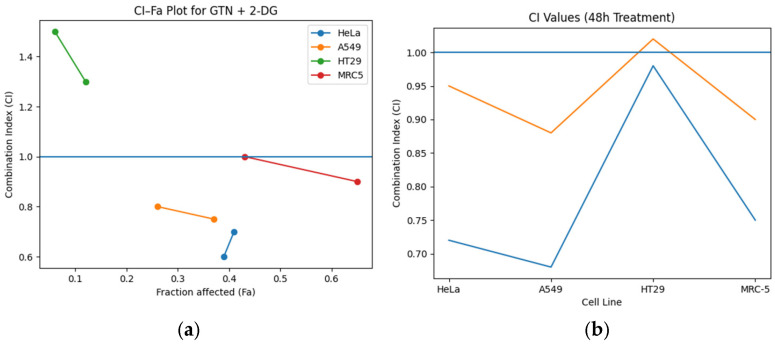
Cell viability following 48 h treatment with nitroglycerin (GTN), 2-deoxy-D-glucose (2-DG), (**a**) HeLa and A549; (**b**) HT29 and MRC-5. Data are presented as mean ± SD.

Overall, the combined treatment enhanced cytotoxicity compared to single agents, particularly in HeLa and A549 cells; however, the reduced selectivity toward normal fibroblasts remains a major limitation for potential therapeutic application. The reduction in cell viability after 48 h treatment is shown in Figure 4.

These findings suggest that GTN contributes to enhanced cytotoxicity under 2-deoxy-D-glucose-induced metabolic stress, while caffeine may further potentiate this response in a cell-type-dependent manner.

## 3. Discussion

The present results demonstrate that nitroglycerin (GTN), although weakly cytotoxic when applied alone, can significantly enhance anticancer effects under conditions of metabolic stress induced by 2-deoxy-D-glucose (2-DG). This observation is consistent with the known requirement for enzymatic activation of GTN to release nitric oxide (NO), since GTN acts as a prodrug whose biological activity depends on mitochondrial aldehyde dehydrogenase (ALDH2)-mediated bioactivation [5,11]. Low cytotoxicity of GTN alone in HeLa and A549 cells may therefore reflect insufficient intracellular NO accumulation under standard conditions. Similar findings have been reported previously, where NO donors showed limited direct cytotoxicity unless combined with additional metabolic or oxidative stressors [3,11].

The enhanced cytotoxicity observed after co-treatment with 2-DG may be explained by the combined effects of glycolytic inhibition and redox imbalance, since increased intracellular ROS production represents one of the major mechanisms of metabolic stress-induced apoptosis in cancer cells [19,20]. Since 2-DG disrupts glycolysis and reduces intracellular ATP production, cancer cells become more vulnerable to oxidative and nitrosative damage induced by NO [5,6]. This is in agreement with previous studies demonstrating that metabolic stress induced by 2-DG sensitizes tumor cells to chemotherapy and promotes apoptosis through energetic collapse and impaired cellular adaptation [5,7]. The strongest effects were observed in HeLa and A549 cells, indicating that these cell lines are particularly sensitive to combined metabolic and oxidative stress.

In contrast, HT29 cells showed weaker responses and predominantly antagonistic effects in CI–Fa analysis, suggesting a greater degree of metabolic adaptability or intrinsic resistance. Colorectal cancer cells are known to exhibit high metabolic plasticity and may rely on alternative pathways such as oxidative phosphorylation, glutamine metabolism, or enhanced antioxidant defense systems, which can reduce sensitivity to glycolysis inhibition [5,6]. This may explain the lower responsiveness of HT29 cells observed in the present study.

Previous studies have shown that caffeine enhances apoptosis through increased ROS production, inhibition of DNA repair pathways, and checkpoint abrogation, thereby sensitizing tumor cells to anticancer treatment [13,14,18]. Caffeine is known to interfere with cell cycle regulation, DNA damage repair pathways, and adenosine receptor signaling, thereby increasing susceptibility to apoptotic stimuli [14,15,17,18]. Previous studies have shown that caffeine can potentiate the cytotoxic effects of chemotherapeutic agents through increased ROS production and inhibition of checkpoint-mediated survival pathways [13,14,18]. The present findings suggest that caffeine may enhance GTN-induced cytotoxicity under metabolically stressed conditions, particularly in A549 cells, where the strongest reduction in viability was observed.

The molecular docking results provide supportive mechanistic insight for these biological observations. Nitroglycerin showed favorable binding to ALDH2 structures 1O01 and 1O02, with binding energies ranging from −6.3 to −6.5 kcal/mol, indicating thermodynamically favorable interactions. The predominance of hydrogen bonding interactions with residues such as Arg84, His156, Tyr139, and Thr143 supports stable ligand positioning within the catalytic region of ALDH2, which is consistent with the established role of this enzyme in GTN bioactivation [4,5]. Similarly, caffeine demonstrated favorable interactions with the adenosine A2A receptor structures 3EML and 5G53 [21], involving hydrogen bonding, aromatic interactions, and hydrophobic stabilization. Since caffeine is a well-known antagonist of A2A receptors, these findings support the biological relevance of the selected targets and confirm the plausibility of receptor-mediated modulation of proliferation and stress signaling in cancer cells [17,22,23,24,25]. The observed docking results indicate that GTN can adopt favorable predicted binding poses within the investigated ALDH2 structures, supporting the structural plausibility of this ligand–target interaction. The observed results indicate a satisfactory binding affinity of GTN toward the investigated proteins, supporting the possibility of biologically relevant interactions. Similarly, caffeine showed favorable predicted interactions with proteins 3EML and 5G53. However, these findings should be considered only supportive theoretical observations, and additional experimental validation would be required to determine whether ALDH2-dependent or adenosine receptor-dependent mechanisms contribute to the cytotoxic responses observed under the conditions investigated.

**Figure 5:**
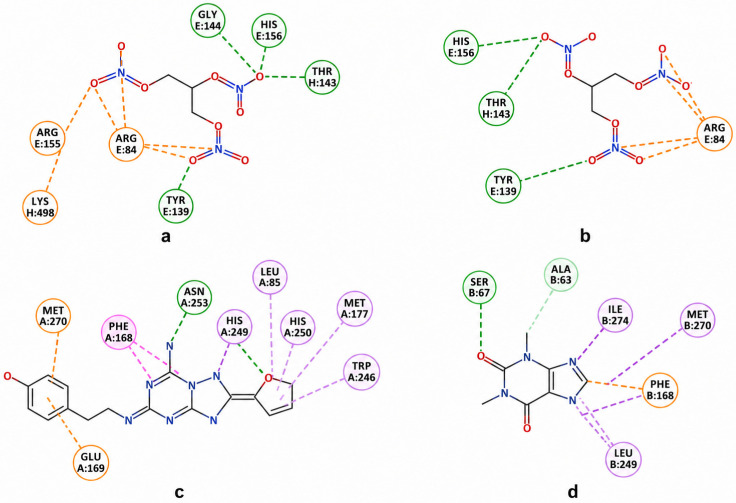
Two-dimensional interaction maps of nitroglycerin and caffeine with the selected protein targets. Nitroglycerin showed predominantly hydrogen-bond-driven binding in ALDH2 structures 1O01 (**a**) and 1O02 (**b**), whereas caffeine formed a combination of hydrogen-bonding, aromatic, and hydrophobic interactions in the adenosine A2A receptor structures 3EML (**c**) and 5G53 (**d**).

Proteins 3EML and 5G53 represent structures of the adenosine A2A receptor, a well-known pharmacological target of caffeine as an antagonist [17,21,23,24,26,27]. The A2A receptor has been implicated in the regulation of proliferation, inflammation, and immune responses in tumors [22,27,28]. Therefore, docking of caffeine to 3EML and 5G53 was included not to identify a novel molecular target of caffeine, but to provide a standardized in silico visualization of caffeine interaction with an established literature-supported target. Due to the experimentally confirmed interaction of caffeine with the A2A receptor and the availability of high-resolution crystal structures [21,24], these proteins were selected as appropriate reference structures for molecular docking (Figure 5). However, the functional relevance of A2A receptor signaling in the investigated cell lines remains to be experimentally confirmed.

An important limitation of this study is the substantial cytotoxicity observed in normal MRC-5 fibroblasts, indicating reduced selectivity of the combined treatment. Although synergistic effects were observed in cancer cells, particularly in HeLa and A549, the pronounced reduction in normal cell viability significantly limits immediate therapeutic applicability. Similar challenges have been reported in studies involving metabolic inhibitors, where enhanced antitumor efficacy is frequently accompanied by toxicity toward non-malignant cells [7]. Therefore, future studies should focus on optimizing dosing regimens, treatment scheduling, and targeted delivery approaches in order to improve selectivity and reduce off-target toxicity.

The CI–Fa analysis provided quantitative confirmation of the interaction between GTN and 2-DG. HeLa cells consistently demonstrated CI values below 1, indicating strong synergistic interactions, while A549 cells showed a shift from synergistic to nearly additive effects at higher fractions affected. In contrast, HT29 cells predominantly exhibited antagonistic interactions (CI > 1), suggesting reduced responsiveness to combined metabolic and oxidative stress. These findings indicate that the therapeutic potential of this combination strongly depends on tumor-specific metabolic characteristics and further emphasize the importance of cellular context in determining treatment efficacy.

Overall, the present study demonstrates that GTN can enhance cytotoxic responses under metabolically stressed conditions induced by 2-DG and further potentiated by caffeine. While the strongest synergistic effects were observed in HeLa and A549 cells, the reduced selectivity toward normal fibroblasts remains a major limitation. Nevertheless, the combination of experimental cytotoxicity assays, CI–Fa analysis, and molecular docking provides a strong basis for further investigation of GTN-based combination strategies in cancer therapy [29,30]. The selectivity index analysis demonstrated moderate tumor selectivity of 2-DG. The highest selectivity was observed in HT29 cells after 24 h treatment (SI = 2.07) and in HeLa cells after 48 h treatment (SI = 2.28). In contrast, A549 cells exhibited an SI value below 1 after 48 h (SI = 0.84), indicating greater susceptibility of normal MRC-5 fibroblasts than tumor cells under these conditions. These findings suggest that the anticancer efficacy of 2-DG is strongly dependent on cell type and exposure time, highlighting the importance of metabolic heterogeneity in determining treatment response (Table S4).

These findings suggest that GTN-based combination strategies may represent a promising approach for enhancing anticancer efficacy under metabolically vulnerable conditions. GTN (nitroglycerin) was selected as the nitric oxide donor in this study due to its documented ability to enhance the efficacy of anticancer therapies, modulate tumor perfusion and hypoxia, and its considerable translational potential in oncology [29,30]. NO has a dual role in tumors, influencing angiogenesis, oxidative stress, and therapeutic response [29].

## 4. Materials and Methods

### 4.1. Cell Lines

Human cervical carcinoma HeLa (ATCC^®^ CCL-2™), A549 lung adenocarcinoma (ATCC^®^ CCL-185™), HT29 colorectal carcinoma (ATCC^®^ HTB-38™), and normal human lung fibroblasts MRC-5 (ATCC^®^ CCL-171™) were obtained from the American Type Culture Collection (ATCC, Manassas, VA, USA). Cells were cultured in Dulbecco’s Modified Eagle Medium (DMEM, 4.5 g/L glucose) supplemented with 10% fetal bovine serum and 1% antibiotic–antimycotic solution.

Cells were maintained at 37 °C in a humidified atmosphere with 5% CO_2_ and used during the exponential growth phase. For experiments, cells were seeded in 96-well plates (1 × 10^4^ cells/well) for viability assays or in 25 cm^2^ flasks (1 × 10^6^ cells/flask).

### 4.2. Reagents

The following reagents were used: 2-deoxy-D-glucose (2-DG) (Abcam, Cambridge, UK), nitroglycerin (5 mg/1.6 mL; Hemofarm, Vršac, Serbia), caffeine (≥99.9%, Abcam, Cambridge, UK), sulforhodamine B (Sigma-Aldrich Chemie GmbH, Taufkirchen, Germany), Tris base (Sigma-Aldrich Chemie GmbH, Germany), trichloroacetic acid (Merck Chemie GmbH, Darmstadt, Germany), acetic acid (Zorka Pharma Hemija, Šabac, Serbia), dimethyl sulfoxide (DMSO), and DMEM (Sigma-Aldrich Chemie GmbH, Germany). All treatment solutions were prepared immediately prior to use. Final DMSO concentration did not exceed 0.1% (*v*/*v*).

### 4.3. Cell Viability and Proliferation Assays

#### 4.3.1. Trypan Blue Exclusion Assay

Only viable cells were used in the experiments. Cell viability was determined using the Trypan Blue exclusion method [31]. Cells were mixed with 0.1% Trypan Blue and counted using a hemocytometer under an inverted microscope.

#### 4.3.2. Sulforhodamine B (SRB) Assay

Cell viability was assessed using the sulforhodamine B (SRB) assay. This colorimetric method is based on the binding of the anionic dye sulforhodamine B to basic amino acid residues of cellular proteins under mildly acidic conditions. The amount of bound dye is proportional to the total cellular protein content and, consequently, to the number of viable cells. Cells were treated with increasing concentrations of nitroglycerin, 2-deoxy-D-glucose (2-DG), and caffeine, individually or in combination, for 24 and 48 h. Following treatment, cells were fixed with trichloroacetic acid, stained with SRB, washed with acetic acid, and the protein-bound dye was solubilized. Absorbance was measured at 540 nm, and cell viability was calculated relative to untreated control cells [32].

### 4.4. Statistical Analysis

All experiments were performed in triplicate, and results are presented as mean ± standard deviation. Statistical significance was determined using one-way ANOVA followed by Tukey’s post hoc test (*p* ≤ 0.05). IC_50_ values were calculated from dose–response curves using nonlinear regression analysis. Drug interactions were evaluated using the Chou–Talalay method [33], and combination index (CI) [34] values were calculated using CalcuSyn software, version 2.1 (Biosoft, Cambridge, UK) based on the median-effect principle to assess synergistic, additive, or antagonistic effects. Statistical analyses were performed using GraphPad Prism version 8.0 (GraphPad Software, San Diego, CA, USA).

### 4.5. Molecular Docking

Molecular docking was performed using the CB-Dock2 platform, which applies a blind docking approach guided by cavity detection to predict potential binding sites and estimate binding affinity. The three-dimensional structures of the target proteins (PDB IDs: 1O01, 1O02, 3EML, and 5G53) were retrieved from the Protein Data Bank.

The 3D structures of the ligands, nitroglycerin and caffeine, were obtained through geometry optimization using the Gaussian 16 program, employing the B3LYP functional with the 6-311G** basis set [35]. The optimized structures were converted into MOL2 format and used as input for docking simulations.

The binding site with the lowest Autodock Vina score was selected as the most favourable interaction model. Binding energies were calculated, and the corresponding binding modes were visualised in a three-dimensional (3D) representation. Two-dimensional (2D) interaction diagrams were generated using Discovery Studio to clearly illustrate hydrogen bonds, hydrophobic contacts, and other relevant molecular interactions. Docking results, including binding energies, ligand binding poses, and interactions with amino acid residues, were analyzed to evaluate the stability of the complexes and the potential therapeutic relevance of the investigated compounds toward the selected protein targets [36].

## 5. Conclusions

The present study demonstrates that metabolic stress induced by 2-deoxy-D-glucose (2-DG) enhances the cytotoxic activity of glyceryl trinitrate (GTN) in cancer cells, with the most pronounced effects observed in HeLa and A549 cell lines. Combination index analysis revealed predominantly synergistic interactions in these cell types, indicating that simultaneous targeting of cellular metabolism and redox homeostasis may represent a promising strategy for increasing anticancer efficacy. In contrast, HT29 cells exhibited lower sensitivity and predominantly antagonistic responses, highlighting the importance of tumor-specific metabolic characteristics in determining treatment outcomes.

The addition of caffeine further potentiated cytotoxic responses under metabolically stressed conditions, particularly after prolonged exposure, suggesting that modulation of stress-response and cell-cycle regulatory pathways may contribute to the observed effects. Molecular docking analyses supported the structural plausibility of GTN interaction with ALDH2 and caffeine interaction with the adenosine A2A receptor; however, these findings should be considered exploratory and hypothesis-generating, as no experimental validation of target engagement or pathway activation was performed.

A major limitation of this study is the substantial cytotoxicity observed in normal MRC-5 fibroblasts, indicating reduced selectivity of the investigated treatment strategy. Furthermore, the absence of apoptosis-specific assays, ROS quantification, and in vivo validation limits mechanistic interpretation and translational relevance. Therefore, future studies should focus on elucidating the molecular mechanisms underlying the observed synergistic effects, improving tumor selectivity, and validating therapeutic efficacy in more physiologically relevant experimental models.

Overall, the findings suggest that the combination of GTN, caffeine, and metabolic stress induced by 2-DG represents a promising approach for exploiting metabolic vulnerabilities in cancer cells and warrants further investigation as a potential anticancer strategy.

## Figures and Tables

**Table 1:** The *IC_50_* value (mM) determined after 24 and 48 h of 2-deoxy-D-glucose (2DG) treatment in *HeLa* (cervical carcinoma), *A549* (lung adenocarcinoma), and *HT29* (colorectal carcinoma).

Cell Line	2 DG 24 h	2DG 48 h
HeLa	5.45	2.01
A549	4.97	5.44
HT29	3.40	4.25
MRC-5	7.05 ^1^	4.58 ^1^

^1^ Abbreviations: normal lung fibroblasts (MRC-5)—control cell line.

**Table 2:** Free energy of binding (kcal/mol).

Proteins	GTN	Caffeine
1O01	−6.5	
1O02	−6.3	
3EML		−6.2
5G53		−6.5

## Data Availability

Data are contained within the article and Supplementary Materials.

## Supplementary Materials

The following supporting information can be downloaded at: https://www.mdpi.com/article/10.3390/molecules31111946/s1, Table S1. Viability after 48 h treatment with GTN + 1 mM 2-DG. Table S2. IC_50_ values of NTG after 24 h and 48 h treatment and NTG in the presence of Caff 1 mM. Table S3. CI–Fa interpretation for GTN + 2-DG after 48 h. Table S4. Selectivity index (SI) ∗ values of 2-deoxy-D-glucose (2-DG) after 24 h and 48 h treatment in relation to MRC-5. Figure S1. Apoptosis on cancer cell line: (a) Immunofluorescence micrograph of early apoptosis, affected by 2DG on cervical adenocarcinoma *HeLa* cells (1000×); (b) Immunofluorescence micrograph of late apoptosis, affected by 2DG on cervical adenocarcinoma *HeLa* cells (400×).
