# Supplementary material for: Glyceryl Trinitrate Enhances Caffeine Cytotoxicity Under Metabolic Stress in Cancer Cells
Source: Molecules. 2026 Jun 4;31(11):1946. doi: 10.3390/molecules31111946 (PMC13257612; doi:10.3390/molecules31111946)
Supplement: Supplementary file 1 [file molecules-31-01946-s001.zip › molecules-4302879-Figure S1.pdf]

SUPPLEMENTARY MATERIAL
FOR

Glyceryl Trinitrate Enhances Caffeine Cytotoxicity under Metabolic Stress in Cancer Cells

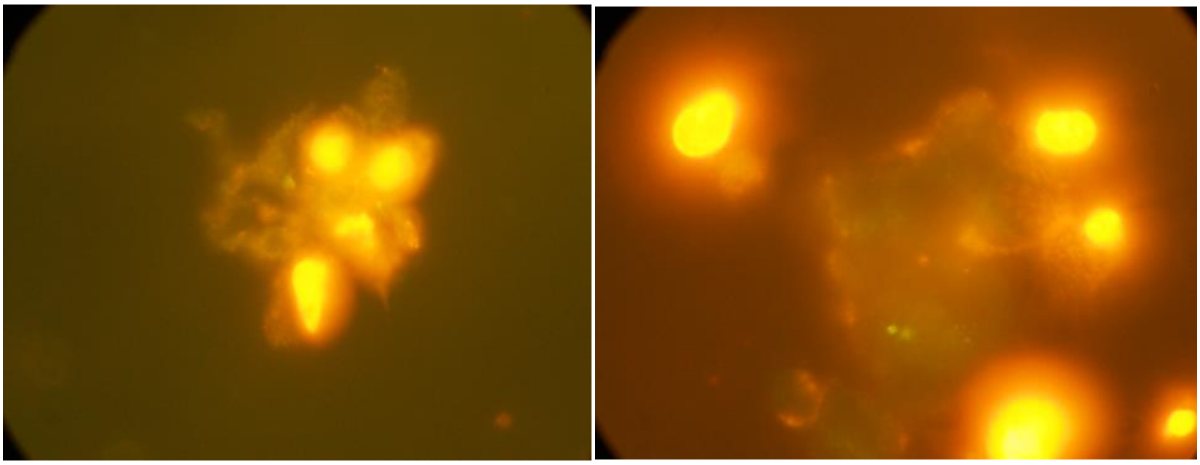

(a) (b)

Figure S1. Apoptosis on cancer cell line: (a) Immunofluorescence micrograph of early apoptosis, affected by 2DG on cervical adenocarcinoma *HeLa* cells (1000x); (b) Immunofluorescence micrograph of late apoptosis, affected by 2DG on cervical adenocarcinoma *HeLa* cells (400x) [1]

[1]Footer The moment of bonding Annexin-V late apoptosis
