# Supplementary material for: Glyceryl Trinitrate Enhances Caffeine Cytotoxicity Under Metabolic Stress in Cancer Cells
Source: Molecules. 2026 Jun 4;31(11):1946. doi: 10.3390/molecules31111946 (PMC13257612; doi:10.3390/molecules31111946)
Supplement: Supplementary file 1 [file molecules-31-01946-s001.zip › molecules-4302879-supplementary.pdf]

SUPPLEMENTARY MATERIAL
FOR

Glyceryl Trinitrate Enhances Caffeine Cytotoxicity under Metabolic Stress in Cancer Cells

Table of Contents

Table S1. Viability after 48 h treatment with GTN + 1 mM 2-DG

Cell line	Most effective combination	Viability after 48 h (%)	Fraction affected, Fa
HeLa	GTN + 1 mM 2-DG	~59–61	~0.39–0.41
A549	10 μM GTN + 1 mM 2-DG	~48.7–62.7	~0.37–0.51
HT29	10 μM GTN + 1 mM 2-DG	~75.9–77.7	~0.22–0.24
MRC-5	10 μM GTN + 1 mM 2-DG	~35–40	~0.60–0.65

Table S2. IC_{50} values of nitroglycerin in the presence of 1 mM caffeine.

Cell line	NTG			NTG + 1 mM Caff	
	IC_{50} (24 h)	IC_{50} (48 h)	Comment	IC_{50} (24 h)	IC_{50} (48 h)
HeLa	ND / >10 μM	ND / >10 μM	IC_{50} out of range	ND (>10 μM)	ND (>10 μM)
A549	ND / >10 μM	ND / >10 μM	ND	2.5 μM	~8–10 μM
HT29	ND / >10 μM	ND / >10 μM	the lowest viability 67.20% at 10 μM	>10 μM	>10 μM
MRC-5	ND / >10 μM	ND / >10 μM	IC_{50} out of range	ND (>10 μM)	ND (>10 μM)

Table S3.CI–Fa interpretation for GTN + 2-DG after 48 h

Cell line	Fa range	CI pattern	Interpretation
**HeLa**	~0.20–0.41	CI < 1	Synergism
**A549**	~0.19–0.40	CI < 1 to ≈1	Synergism to nearly additive
**HT29**	~0.20–0.30	CI > 1	Antagonism
**MRC-5**	Variable	variable CI	Non-selective cytotoxicity / variable interaction

Table S4. Selectivity index (SI)* values of 2-deoxy-D-glucose (2-DG) after 24 h and 48 h treatment in relation to MRC-5

Cell line	SI (24 h)	SI (48 h)
**HeLa**	1.29	2.28
**A549**	1.42	0.84
**HT29**	2.07	1.08

***SI = IC_{50} (MRC-5) / IC_{50} (tumor cell line). Interpretation:** SI values > 1 indicate preferential cytotoxicity toward cancer cells relative to normal fibroblasts, whereas SI values < 1 indicate greater sensitivity of normal cells. The highest selectivity of 2-DG was observed in HT29 cells after 24 h treatment (SI = 2.07) and in HeLa cells after 48 h treatment (SI = 2.28), while A549 cells exhibited poor selectivity after 48 h (SI = 0.84).
